# Influence of Statins locally applied from orthopedic implants on osseous integration

**DOI:** 10.1186/1471-2474-13-208

**Published:** 2012-10-26

**Authors:** Stephan Pauly, David A Back, Kathrin Kaeppler, Norbert P Haas, Gerhard Schmidmaier, Britt Wildemann

**Affiliations:** 1 Julius Wolff Institut, Center for Musculoskeletal Surgery, Charité Universitaetsmedizin Berlin, Berlin, Germany; 2 Berlin-Brandenburg Center for Regenerative Therapies, Charité Universitaetsmedizin Berlin, Berlin, Germany; 3 Department of Orthopedics and Traumatology, German Armed Forces Hospital Berlin, Berlin, Germany; 4 Department of Orthopedic and Trauma Surgery, University of Heidelberg, Heidelberg, Germany

**Keywords:** Statins, Simvastatin, Implant integration, BMP, Local application

## Abstract

**Background:**

Simvastatin increases the expression of bone morphogenetic protein 2 (BMP-2) in osteoblasts, therefore it is important to investigate the influence of statins on bone formation, fracture healing and implant integration. The aim of the present study was to investigate the effect of Simvastatin, locally applied from intramedullary coated and bioactive implants, on bone integration using biomechanical and histomorphometrical analyses.

**Methods:**

Eighty rats received retrograde nailing of the femur with titanium implants: uncoated vs. polymer-only (poly(D,L-lactide)) vs. polymer plus drug coated (either Simvastatin low- or high dosed; “SIM low/ high”). Femurs were harvested after 56 days for radiographic and histomorphometric or biomechanical analysis (push-out).

**Results:**

*Radiographic* analysis revealed no pathological findings for animals of the control and SIM low dose group. However, n=2/10 animals of the SIM high group showed osteolysis next to the implant without evidence of bacterial infection determined by microbiological analysis. *Biomechanical* results showed a significant decrease in fixation strength for SIM high coated implants vs. the control groups (uncoated and PDLLA). *Histomorphometry* revealed a significantly reduced total as well as direct bone/implant contact for SIM high- implants vs. controls (uncoated and PDLLA-groups). Total contact was reduced for SIM low vs. uncoated controls. Significantly reduced new bone formation was measured around SIM high coated implants vs. both control groups.

**Conclusions:**

This animal study suggests impaired implant integration with local application of Simvastatin from intramedullary titanium implants after 8 weeks when compared to uncoated or carrier-only coated controls.

## Background

The increasing number of primary and revision surgeries following orthopaedic implant application suggests the need for a durable osseous integration. Optimal osseointegration depends on the formation of new bone around orthopaedic implants, which may be stimulated by osteoinductive agents.

One safe [1] and promising substance group for this aim are the statins, where were primarily developed to inhibit cholesterol biosynthesis [2] and reduce its plasma levels [3]. Besides other pleiotropic effects [4,5], Simvastatin furthermore stimulates bone formation in a human osteoblast cell line (MG-63) via increased expression of bone morphogenetic protein (BMP-2) [6]. BMP-2 is one of the most potent growth factors targeting bone formation in vivo [7], which stimulates osteoblast differentiation and -proliferation [8-10].

With systemic [11-14] or local delivery of statins to the bone from biodegradable carriers or percutaneous application forms, bone formation and fracture healing were improved in several experimental investigations [15-22].

Statins were also shown to improve implant integration following arthroplastic surgery. Although there have been multiple studies using systemic administration [11,23-25], only a few studies have investigated local application of statin-containing gels or microspheres and their effect on implant-bone integration [26-28]. In summary, combining the bone anabolic effect of Simvastatin with other local drug application techniques is a promising approach. Local application seems to be a reasonable strategy, as statins target liver metabolism and are poorly distributed to bone due to first pass elimination [29]. Furthermore, local application helps prevent systemic side effects of drugs.

Hence, it was the aim of the present study to investigate the effect of Simvastatin, locally applied in a biodegradable Poly(D,L-lactide) coating from intramedullary implants [30], on bone integration using biomechanical and histomorphometrical analyses.

We hypothesized that Simvastatin would improve histomorphometrical and biomechanical implant integration when compared to uncoated or carrier only-coated devices.

## Results

There were no differences in body temperature and -weight between the groups over the experimental time period. None of the animals showed evidence of local or systemic infection throughout the experimental period.

**Figure 1:**
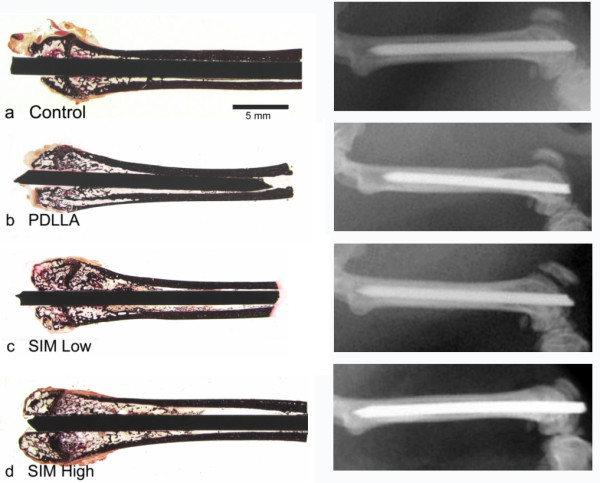
**Histomorphometric overview and x-ray after 56 days, for animals from a) uncoated group, b) PDLLA, c) SIM low and d) SIM high.** No macroscopic differences were obvious between all 4 groups with regard to implant integration, osteolysis, ectopic bone formation or implant loosening.

**Figure 2:**
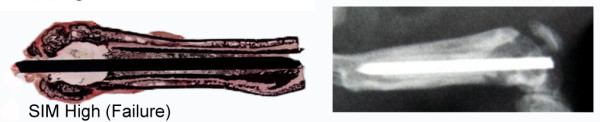
**Microscopic and radiographic findings in one (out of n=2/20) SIM high-treated femur after 56 days, showing osteolysis around the implant.** Bacterial infection was ruled out by means of microbiological diagnostics.

After 56 days at the *x-ray control*, no radiographic implant dislocation was observed (Figure 1). However, n=2/20 animals of the SIM high-group showed osteolysis around the implants (Figure 2) and n=1/20 animal from both the PDLLA and SIM high group had broadened medullary canals. However, *microbiological analysis* of these affected femurs showed no evidence of bacterial infections.

**Figure 3:**
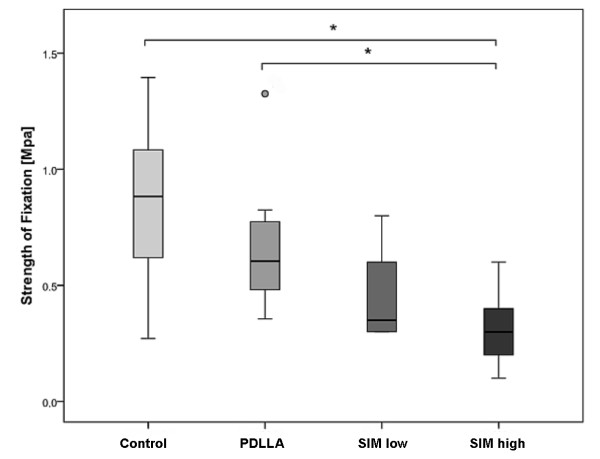
Biomechanical fixation strength of SIM high coated implants was significantly weaker than with uncoated- (p = 0.002) or PDLLA-coated implants (p = 0.005) (*).

Calculated *strength of fixation* was highest for uncoated implants when compared to all other control and experimental groups. Fixation of SIM high coated implants was significantly weaker than with uncoated- (p = 0.002) or PDLLA-coated implants (p = 0.005, Figure 3).

**Figure 4:**
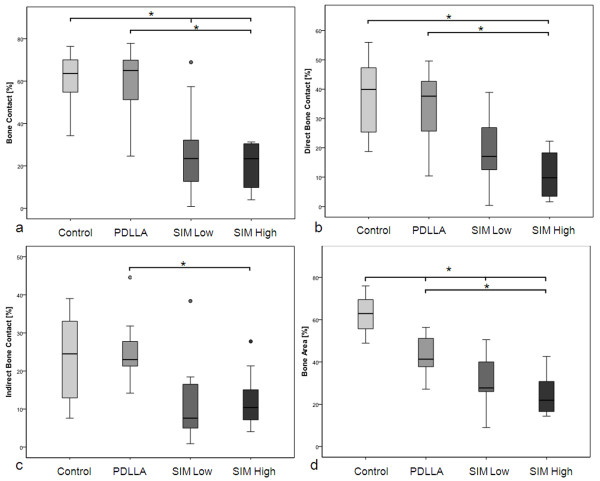
**a) Histomorphometric analysis.** Total bone/implant contact was significantly decreased between the uncoated control and both experimental groups (SIM high: p < 0.001; SIM low: p = 0.006) as well as between PDLLA and SIM high (p < 0.001) (*). **b**) Direct bone/implant contact showed significant differences between SIM high and both control groups (vs. uncoated: p = 0.001; vs. PDLLA: p = 0.006) (*). **c**) Indirect bone/implant contact was reduced for both SIM coated groups, differing significantly between the PDLLA and SIM high group (p = 0.004) (*). **d**) Newly formed bone (within a 0.3mm range around the implants) was significantly reduced between uncoated controls and all other groups (vs. PDLLA: p = 0.002; vs. SIM low and –high: p < 0.001, respectively), as well as between PDLLA and SIM high implants (p = 0.002) (*).

Histomorphometrical analysis with regard to *total bone/implant contact* showed only marginal differences between the control groups (uncoated, PDLLA-coated, Figure 4). However, lower results were detected for both experimental groups, reaching level of significance for SIM high when compared to both control groups (minus 46.0% and 32.8%, respectively, p < 0.001) and for SIM low vs. uncoated controls (p = 0.006).

Neither direct nor indirect bone/implant contact differed significantly between uncoated and PDLLA-coated implants. In terms of *direct contact*, SIM high results were significantly decreased when compared to both control groups (vs. uncoated: p = 0.001; vs. PDLLA: p = 0.006; Figure 4).

*Indirect contact* was highest in the uncoated and PDLLA-coated groups. Both SIM coated groups had lower values than the controls, differing significantly between the PDLLA and SIM high group (minus 51.0%; p = 0.004, Figure 4).

Assessment of *newly formed bone* around the implants revealed significant reduction for the groups PDLLA (p = 0.002) and SIM low and -high (p < 0.001, respectively) when compared to uncoated controls (Figure 4). SIM high implants showed significantly less surrounding new bone than PDLLA-coated implants (p = 0.002).

## Discussion

The present findings suggest impaired implant integration with local application of Simvastatin from intramedullary titanium implants after 8 weeks when compared to uncoated or carrier-only coated controls.

Though the two cases of radiographic osteolysis around the implant occured in the histomorphometric group, biomechanical stability was significantly weaker for SIM high when compared to uncoated and carrier-only coated implants. Histomorphometry confirmed a significant reduction in total bone/implant contact, (in-)direct contact and new bone formation for the experimental groups when compared to the control groups.

In summary, the hypothesis of the present paper with regard to improved implant integration must be rejected for both experimental groups.

The question arises why bone integrational processes deteriorated under Simvastatin exposure when compared to controls. One potential obvious explanation, *intramedullary infections* of the femurs, was ruled out since no bacteria were identified after microbiological analysis over 14 days.

Another possible reason for the adverse effects might be the *PDLLA-coating* itself. However, this polymer was previously shown to be biocompatible, mechanically stable [30] and a reproducibly degradable carrier to locally deliver agents to the bone, without evidence of osteolysis [20,31-34]. Furthermore, the PDLLA-group showed (significantly) superior biomechanical, histomorphometric and radiographic properties when compared to the experimental groups. Thus, PDLLA is likely not the reason for the deteriorated bone integration, even though its biomechanical stability was slightly inferior when compared to uncoated controls.

Another possible reason for the lack of osseointegration may have been the *drug dose* used, since dose-dependent effects of statins on bone metabolism were suggested due to differing sensitivity of osteoblasts and osteoclasts. Bone resorption and formation were elevated with high-dose Simvastatin while low-dose SIM decreased formation and increased bone resorption [35]. The present results seem contrary since SIM high rather than SIM low had bone catabolic effects and neither exerted bone anabolic effects. The dose-dependent, bone-anabolic effect of a comparable SIM high-dose was previously shown to have a similar effect to that of BMP-2 in a rat fracture model [20]. Additionally, one other failure option is the *type of the incorporated drug*. However, the identical substance and coating technique were successfully investigated previously [20]. Several other experimental studies confirmed beneficial effects of statins on *fracture healing* using different local application approaches [18,19,21,22]. In addition, statins improved defect regeneration when locally applied in cranial/mandibular *bone defect* models without metal implants [15-17,36]. These studies used absorbable collagen and gelatin sponges or injections for drug delivery which are prone to dissolve at the site of application. A femoral defect model with local small molecule drug delivery (but no metal implant) revealed bisphosphonates significantly improved bone formation while lovastatin did not [37]. Piskin et al. demonstrated Simvastatin-loaded electrospun nanofibers enhanced bone mineralization (histological and micro-CT analysis) [38]. Even though the same drug was used, the different dosage and the use of the polymer caprolactone represent different approaches than in the present setting, hence impede comparability.

In contrast to fracture- and bone defect healing, *implant integration* was investigated presently. In this regard, different studies investigated the effect of *local statin application*. A similar rodent model was utilized by Moriyama et al. who observed improved tibial implant integration dose-dependently after 7–14 days of local fluvastatin-release from a PGA-coating [26]. In contrast to the present findings, their higher-dosed group (2.5 mg/ml) showed the best results in terms of bone formation and push-out strength. The short observational period of 7 or 14 days may be one reason for different results when compared to the present 56 day period. Further distinctions to the present study are the chosen type of carrier and the incorporated drug Fluvastatin (vs. Simvastatin), although both are lipophilic, penetrate cell membranes and enhance osteogenesis. These authors later investigated injectable PGA-gel around tibia implants in rodents and found similar results [28]. They observed a significant decrease in implant integration at one week comparable to the present results after 56 days. However, stability recovered and significantly increased after 14 and 28 days, respectively. Nevertheless, injected gel or mobile nanoparticles may dissolve from the intramedullary destination, while solid bioactive implant coatings may reduce this effect.

Other experimental studies reported on improved orthopedic implant integration in animals even under *systemical exposure* to statins, administered orally [23], percutaneously [24,27] or intraperitoneally [11,25]. Effects were observed with up to 10-50 mg/kg bodyweight, far exceeding the statin dose rates applied in humans, while the equivalent dose used in humans was ineffective [25]. Hence, systemical application does not seem to be useful for improved implant integration with normal human drug doses. Even though one observational clinical study reported on reduced risk of hip implant revision, deep infection and aseptic loosening among statin users under normal dose rates [39], no direct conclusion on the drug was feasible. It was suggested that statin-users in general might show a more health oriented behaviour (i.e. medication or rehabilitation compliance). One prospectively randomized clinical study found no effect on bone healing between low Simvastatin-intake (20 mg/d orally) and placebo [40].

Since less than 5% of an oral statin dose reaches the circulation due to hepatic first pass elimination [29], systemical application requires rather high drug doses. Targeted, local application of drugs from bioactive carrier polymers seems more efficient and may help to improve drug availability within the bone while lowering necessary drug doses, hence preventing systemic side effects.

As another potential limitation of this study, the *observation period* of eight weeks may be inappropriate to observe differences in implant integration since other authors observed effects of statins with this regard after 7–30 days [11,25-28]. During fracture healing, beneficial effects were reported after 5–14 days of local statin exposure to the fracture site [18,21,22], suggesting that statins cause a delayed onset of endogenous BMP-2 production [20]. Mundy et al. reported on a quick BMP-2 response to statins within 3–5 days in vivo and in vitro [6] while other authors found improved implant integration after 42–84 days following high systemical doses of Simvastatin [23,24].

After 8 weeks, remodeling “back to normal” may occur and initial improvements in implant integration may vanish over time. However, mid- and long term data are important with regard to prosthetic implant integration in humans.

Nevertheless, the timepoint does not explain the osteolysis in two SIM high coated animals.

Even though the current results are discouraging with regard to Simvastatin, local application via biocompatible, stable drug-delivering polymers [30] seems beneficial since no manipulations or injections to the bone are necessary. Previous studies revealed that incorporation of several bioactive agents (such as BMP-2, Zolendronate, Simvastatin) into the PDLLA-coating of bone implants improves fracture healing and implant integration experimentally [20,31-34].

## Conclusions

This animal study suggests impaired implant integration under local application of Simvastatin from intramedullary titanium implants after 8 weeks when compared to uncoated or carrier-only coated controls.

## Methods

### Coating technology and growth factors

Titanium Kirschner wires (“K-wires”, Diameter 1.4 mm) were coated with Poly(D,L-lactide), PDLLA (Boehringer, Ingelheim, Germany), differing in the substance incorporated: uncoated, PDLLA, PDLLA with low-dose Simvastatin (5.5 μg/implant, SIM low) or with high-dose Simvastatin (90 μg/implant, SIM high). A dip coating technique was utilized which was described in more detail earlier [30].

### Animals and surgery

Approval was obtained from the local Animal Experimentation Ethics Committee. According to the 3R concept [41], animals of the control groups (uncoated and PDLLA) were shared with another experimental osseointegration study (data not shown here) in order to reduce the total number of animals.

Eighty female Sprague–Dawley-Rats (mean age: 6 months, mean body weight: 260 g; Harlan-Winkelmann, Germany) were used in the experiments. Twenty animals were randomly assigned to each of the four study groups (n=10 specimen for biomechanical and histomorphometric analysis, respectively).

Animals were sedated with Isoflurane (Forene) and intraperitoneal anesthesia using a mixture of ketamine hydrochlorid (100 mg/mL; 80 mg/kg body weight) and 2% xylazine (12 mg/kg body weight).

After shaving the right hind leg, a 3 mm longitudinal incision was made. Subsequently, the intercondylar notch of the femur was opened carefully with a 1.2 mm hand drill and the respective implant was inserted into the medullar cavity in a retrograde direction. The aimed proximal position of the implant next to the greater trochanter was controlled via fluoroscopy. Finally, the remaining extending part of the implant was cut and skin was sutured.

With regard to pain relief, all rats received buprenorphine (Temgesic®, 0.05 mg/kg) subcutaneously for two days.

Wounds were assessed daily for clinical signs of infections. Body weight and temperature were documented during the experimental period. Digital radiographs in two planes were obtained at the time of surgery and euthanasia.

After 56 days, animals of all groups were sacrificed. The operated femurs were dissected of soft tissues and ten femurs per group were submitted for either biomechanical or histological analysis. Bones presenting with macroscopic or radiographic signs of infection (i.e. redness, swelling or secretion around the femoral approach; osteolysis around the implant) underwent sterile swabs with subsequent culture on blood agar plates. Each 24 hours of incubation time, plates were analyzed for growth of either aerobic or anaerobic bacteria over 14 days.

### Biomechanical testing

Implants were exposed 3 mm proximally and distally in each femur after careful preparation and then embedded with bone cement (Heraeus-Kulzer, Wehrheim, Germany) in a push-out device [32]. Subsequently, a material testing device (Zwick 1455, Ulm, Germany) applied a constant linear propulsion (v = 2 mm/min) to the implanted K-wire. The peak force to loosen the implant was documented for calculating the implant-bone strength. This maximum force was normalized to the total bone-implant contact area in order to minimize variation between push-out forces due to varying bone or implant length [32]: Strength of fixation σ_u_ = F_max_/πDH [σ_u_: strength of fixation (Mpa), F_max_: initial push-out force (N), D: implant diameter (mm), H: Bone length (mm)].

### Histomorphometry

After fixation of the bones in 10% normal buffered formaldehyde for 2 days, dehydration in ascending concentrations of ethanol and undecalcified embedding in methylmethacrylate (Technovit 7200, Heraeus, Wehrheim, Germany) followed.

**Figure 5:**
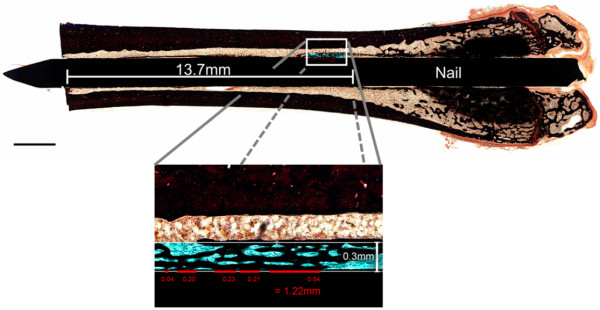
**Histological samples were analyzed following a 13.7 mm line (from the nutrient foramen to the distal femur) to determine bone/implant contact and new bone formation, within a 0.3 mm region of interest, on both sides of the implant**[42].

Specimens were ground (Exakt, Norderstedt, Germany) until implants appeared in full length with maximum diameter. The bottom areas were glued to microscope slides, cut into 300 μm sections with a diamond band saw (Exakt, Norderstedt, Germany) and then ground to 80 μm. Different stainings were used, including Safranin-O and silvering/van Kossa (for mineralized tissues). Histological parameters were assessed using an image analysis system (Axioskop 40, Carl Zeiss, Goettingen, Germany). Within the region of interest (ROI, definition: virtual line of 13.7 mm starting from the nutrient foramen [42]), direct and indirect bone/implant contact were determined with an analyzing software (AxioVision 4.7, Carl Zeiss, Jena, Germany). Direct bone contact was defined as trabecular bone adjacent to the implant surface and indirect contact when a gap existed between the implant and bone. The ratio of direct-, indirect and total bone/implant contact of both cortices within the ROI was calculated. Finally, new bone formation was determined as trabecular mass within a 0.3 mm region on both sides of the implant inside of the ROI, normalized to the total space of this area (Figure 5). 

### Statistics

All animals were randomized in a blinded manner for histological or biomechanical investigation. Comparison of biomechanical and histomorphometrical data was performed using Kruskal-Wallis and Mann–Whitney Test. Tests were controlled for multiple comparison using Bonferroni-Holm correction. Statistical differences were defined at the 95% confidence level. Statistical software (SPSS release 14.0, SPSS, Inc., Chicago, IL) was used for evaluation.

## Abbreviations

BMP: Bone Morphogenetic Protein; PDLLA: Poly(D,L-lactide); ROI: Region of interest; SIM: Simvastatin.

## Competing interests

All authors confirm that there are no conflicts of interest associated with this publication and there has been no financial support for this work that could have influenced its outcome. Financial support was provided by non-profit organizations exclusively (BMBF, Berlin Brandenburg Center for Regenerative Therapies/BCRT).

We declare that this manuscript is original, has not been published before and is not currently being considered for publication elsewhere. It has been read by all authors and each of the authors is convinced that the manuscript represents honest work.

## Authors’ contributions

SP participated in animal experiments, analyzed data and wrote the manuscript. DB carried out all animal operations and participated in the manuscript. KK performed histologic and biomechanical testings and statistical analysis. NH conceived of the study. GS conceived of the study, and participated in its design and coordination. BW conceived of the study, developed its design and critically revised the manuscript. All authors read and approved the final manuscript.

## Pre-publication history

The pre-publication history for this paper can be accessed here:

http://www.biomedcentral.com/1471-2474/13/208/prepub
